# Supplementary material for: Branched chain amino acids harbor distinct and often opposing effects on health and disease
Source: Commun Med (Lond). 2023 Nov 28;3:172. doi: 10.1038/s43856-023-00382-x (PMC10684599; doi:10.1038/s43856-023-00382-x)
Supplement: Supplementary file 1 — Description of Supplemental Files [file 43856_2023_382_MOESM1_ESM.docx]

**Description of additional supplementary files**

Supplementary Data 1. Demographic and clinical characteristics of 97,469 European ancestry UK Biobank participants at study baseline (2006-2010).

Supplementary Data 2. Partial r for measured and genetically inferred leucine (Leu), isoleucine (Ile), and valine (Valine) in n=97,468 UK Biobank participants of European ancestry at study baseline (2006-2010).

Supplementary Data 3. Genome wide-significant (P< 1.7x10-9) independent signals discovered in three GWAS of branched chain amino acids isoleucine, leucine, and valine in n= 97,469 European ancestry UK Biobank participants.

Supplementary Data 4. Estimated narrow-sense heritability and PRS performance R2 (%) estimated from a genome-wide association study of n=97,469 European ancestry UK Biobank participants.

Supplementary Data 5. Phenotype characteristics, presented as mean (standard deviation, SD) or proportion for a maximum of N=97,469 European ancestry UK Biobank participants.

Supplementary Data 6. BCAA effects for 441 phenotypes estimated with (multivariable Mendelian randomization) and without (univariable Mendelian randomization) accounting for the correlation between BCAAs. Association results with measured BCAAs are also presented to enable comparisons with published observational studies. Effects for binary phenotypes are presented as log effect estimates.

Supplementary Data 7. Description of investigator-measured continuous and categorical phenotypes assessed in a maximum of N=97,469 UK Biobank European ancestry participants.

Supplementary Data 8. Description of inpatient phenotypes and phecodes constructed in a maximum of N=97,469 UK Biobank European ancestry participants.

Supplementary Data 9. Individual F statistics and Sanderson-Windmeijer F-statistics evaluating instrument strength for continuous phenotypes.
