# Supplementary material for: Branched chain amino acids harbor distinct and often opposing effects on health and disease
Source: Commun Med (Lond). 2023 Nov 28;3:172. doi: 10.1038/s43856-023-00382-x (PMC10684599; doi:10.1038/s43856-023-00382-x)
Supplement: Supplementary file 3 — Reporting Summary [file 43856_2023_382_MOESM3_ESM.pdf]

nature portfolio

Corresponding author(s): Christy Avery

Last updated by author(s): Apr 11, 2023

Reporting Summary

Nature Portfolio wishes to improve the reproducibility of the work that we publish. This form provides structure for consistency and transparency in reporting. For further information on Nature Portfolio policies, see our Editorial Policies and the Editorial Policy Checklist.

Statistics

For all statistical analyses, confirm that the following items are present in the figure legend, table legend, main text, or Methods section.

n/a	Confirmed	
☐	☒	The exact sample size (*n*) for each experimental group/condition, given as a discrete number and unit of measurement
☐	☒	A statement on whether measurements were taken from distinct samples or whether the same sample was measured repeatedly
☐	☒	The statistical test(s) used AND whether they are one- or two-sided<br>*Only common tests should be described solely by name; describe more complex techniques in the Methods section.*
☐	☒	A description of all covariates tested
☐	☒	A description of any assumptions or corrections, such as tests of normality and adjustment for multiple comparisons
☐	☒	A full description of the statistical parameters including central tendency (e.g. means) or other basic estimates (e.g. regression coefficient) AND variation (e.g. standard deviation) or associated estimates of uncertainty (e.g. confidence intervals)
☐	☒	For null hypothesis testing, the test statistic (e.g. *F*, *t*, *r*) with confidence intervals, effect sizes, degrees of freedom and *P* value noted<br>*Give P values as exact values whenever suitable.*
☒	☐	For Bayesian analysis, information on the choice of priors and Markov chain Monte Carlo settings
☒	☐	For hierarchical and complex designs, identification of the appropriate level for tests and full reporting of outcomes
☐	☒	Estimates of effect sizes (e.g. Cohen's *d*, Pearson's *r*), indicating how they were calculated

Our web collection on statistics for biologists contains articles on many of the points above.

Software and code

Policy information about availability of computer code

Data collection	No open source or custom code was used for data collection.
Data analysis	No open source or custom code was used for data analysis.

For manuscripts utilizing custom algorithms or software that are central to the research but not yet described in published literature, software must be made available to editors and reviewers. We strongly encourage code deposition in a community repository (e.g. GitHub). See the Nature Portfolio guidelines for submitting code & software for further information.

Data

Policy information about availability of data

All manuscripts must include a data availability statement. This statement should provide the following information, where applicable:

- Accession codes, unique identifiers, or web links for publicly available datasets
- A description of any restrictions on data availability
- For clinical datasets or third party data, please ensure that the statement adheres to our policy

All data are publicly available through the UK Biobank (https://biobank.ndph.ox.ac.uk/).

Human research participants

Policy information about studies involving human research participants and Sex and Gender in Research.

Reporting on sex and gender	Findings applied to one sex when appropriate, e.g., when associations with ovarian cancer or prostate cancer were examined.
Population characteristics	Please see manuscript: A total of 97,469 participants of European ancestry were included. At study baseline, participants were on average middle age (mean age = 56.7 years) and female (54.1. Approximately 67% of participants were classified as overweight or obese (mean body mass index = 27.4) and 4.6% of participants were classified as having type 2 diabetes.
Recruitment	Please see manuscript: The United Kingdom Biobank (UK Biobank) is a publicly available, national longitudinal study of UK residents 36. As described previously, 9.2 million men and women aged 40-69 years registered with the National Health Service were invited to attend one of 22 assessment centers located in England, Scotland, and Wales. Of these eligible participants, 502,464 participants (5.5%) enrolled in the study.
Ethics oversight	Please see manuscript: This study was approved by the institutional review board of the University of North Carolina at Chapel Hill.

Note that full information on the approval of the study protocol must also be provided in the manuscript.

Field-specific reporting

Please select the one below that is the best fit for your research. If you are not sure, read the appropriate sections before making your selection.

☒ Life sciences ☐ Behavioural & social sciences ☐ Ecological, evolutionary & environmental sciences

For a reference copy of the document with all sections, see nature.com/documents/nr-reporting-summary-flat.pdf

Life sciences study design

All studies must disclose on these points even when the disclosure is negative.

Sample size	As described in the manuscript, we included n=97,469 participants of European ancestry with measured BCAAs and genotypic data.
Data exclusions	Due to small numbers, we excluded participants of non-European ancestry. We also excluded participants without measured BCAAs and genotypic data.
Replication	We did not seek replication.
Randomization	Randomization was not performed.
Blinding	As this is an observational study, blinding was not performed.

Reporting for specific materials, systems and methods

We require information from authors about some types of materials, experimental systems and methods used in many studies. Here, indicate whether each material, system or method listed is relevant to your study. If you are not sure if a list item applies to your research, read the appropriate section before selecting a response.

Materials & experimental systems

n/a	Involved in the study	
☒	☐	Antibodies
☒	☐	Eukaryotic cell lines
☒	☐	Palaeontology and archaeology
☒	☐	Animals and other organisms
☒	☐	Clinical data
☒	☐	Dual use research of concern

Methods

n/a	Involved in the study	
☒	☐	ChIP-seq
☒	☐	Flow cytometry
☒	☐	MRI-based neuroimaging
